# Soil and foliar selenium application: Impact on accumulation, speciation, and bioaccessibility of selenium in wheat (*Triticum aestivum* L.)

**DOI:** 10.3389/fpls.2022.988627

**Published:** 2022-09-14

**Authors:** Min Wang, Fei Zhou, Nan Cheng, Ping Chen, Yuanzhe Ma, Hui Zhai, Mingxing Qi, Nana Liu, Yang Liu, Li Meng, Gary S. Bañuelos, Dongli Liang

**Affiliations:** ^1^College of Natural Resources and Environment, Northwest A&F University, Yangling, Shaanxi, China; ^2^State Key Laboratory of Pollution Control and Resource Reuse, School of the Environment, Nanjing University, Nanjing, Jiangsu, China; ^3^Key Laboratory of Oasis Ministry of Education, College of Ecology and Environment, Xinjiang University, Urumqi, China; ^4^Center of Regional Watershed Environment Comprehensive Control Technology in Jiangsu Province, Academy of Environmental Planning & Design, Co., Ltd, Nanjing University, Nanjing, China; ^5^School of Arts, Ankang University, Ankang, Shaanxi, China; ^6^USDA, Agricultural Research Service, San Joaquin Valley Agricultural Sciences Center, Parlier, CA, United States; ^7^Key Laboratory of Plant Nutrition and the Agri-environment in Northwest China, Ministry of Agriculture, Yangling, Shaanxi, China

**Keywords:** bioaccessibility, wheat, flour yield, selenium speciation, selenate, selenite

## Abstract

A comprehensive study in selenium (Se) biofortification of staple food is vital for the prevention of Se-deficiency-related diseases in human beings. Thus, the roles of exogenous Se species, application methods and rates, and wheat growth stages were investigated on Se accumulation in different parts of wheat plant, and on Se speciation and bioaccessibility in whole wheat and white all-purpose flours. Soil Se application at 2 mg kg^–1^ increased grains yield by 6% compared to control (no Se), while no significant effects on yield were observed with foliar Se treatments. Foliar and soil Se application of either selenate or selenite significantly increased the Se content in different parts of wheat, while selenate had higher bioavailability than selenite in the soil. Regardless of Se application methods, the Se content of the first node was always higher than the first internode. Selenomethionine (SeMet; 87–96%) and selenocystine (SeCys_2_; 4–13%) were the main Se species identified in grains of wheat. The percentage of SeMet increased by 6% in soil with applied selenite and selenate treatments at 0.5 mg kg^–1^ and decreased by 12% compared with soil applied selenite and selenate at 2 mg kg^–1^, respectively. In addition, flour processing resulted in losses of Se; the losses were 12–68% in white all-purpose flour compared with whole wheat flour. The Se bioaccessibility in whole wheat and white all-purpose flours for all Se treatments ranged from 6 to 38%. In summary, foliar application of 5 mg L^–1^ Se(IV) produced wheat grains that when grounds into whole wheat flour, was the most efficient strategy in producing Se-biofortified wheat. This study provides an important reference for the future development of high-quality and efficient Se-enriched wheat and wheat flour processing.

## Highlights

- Foliar and soil Se application accumulated higher Se in different parts of wheat.
- first node plays an important role in transferring Se from xylem to phloem.
- SeMet (87–96%) and SeCys_2_ (4–13%) were the main Se species in wheat grains.
- White all-purpose flours caused 12–68% Se lost compared with whole wheat flour.
- Bioaccessibility of different flour extraction rate (70 and 100%) was 6–38%.

## Introduction

Selenium (Se) is an indispensable component of more than 25 Se-containing proteins involved in vital metabolic processes, such as metabolic enzyme thioredoxin reductase (TrxR), iodothyronine deiodinases (DIO), and antioxidant glutathione peroxidase (GSH-Px; Dong et al., 2020). It was estimated that at least one billion people have insufficient Se intake in the world (Zhang et al., 2019), which may cause various diseases in humans, including hypothyroidism, susceptibility to infection, tumors, rheumatoid arthritis, or heart failure (Rayman, 2012). The recommended daily dietary intake of Se generally ranges 50–55 μg day^–1^ (WHO, 2004). Considering that organisms can’t synthesize Se autonomously, human Se intake is primarily from the dietary diet, and food chains strategies to improve Se content in food crops can be achieved through Se biofortification practices (D’Amato et al., 2020; Wang et al., 2021; Zhou F. et al., 2021). Up to now, research on Se biofortification has been conducted in different plants including potato (Zhang et al., 2019; Dong et al., 2021), mushroom (Dong et al., 2020; Zhou et al., 2020), maize (Muleya et al., 2021), wheat (Liang et al., 2020; Xia et al., 2020; Wang et al., 2021), and rice (D’Amato et al., 2018).

Currently, soil and foliar Se application are widely used due to their simplicity and practicability (Dinh et al., 2019; Wang et al., 2020). In general, for soil Se application, there are interactions between the soil and Se, before it is absorbed by plant roots, and transported through xylem to storage parts, leaves, and subsequently to grains, i.e., wheat, via phloem (Li et al., 2008; Ducsay et al., 2016; Gupta and Gupta, 2017; Wang et al., 2019). Selenium can also enter the leaves after foliar Se application by penetrating through the cuticle or via the stomatal pathway (Saha et al., 2017). It is then transported to the edible parts of plant but its re-translocation relies on the nutritional status and phenological stage of plant (Saha et al., 2017; Connor et al., 2018). In cereal crops like wheat, the maturity of leaves determines whether a leaf competes with grain as a sink of Se or whether it can act as a source for Se translocation to grains. Mature leaves can only transport Se directly via phloem to grains but can’t import Se (Saha et al., 2017). Thus, both soil and foliar Se application methods may enhance the transport of Se to the edible parts of plants (Boldrin et al., 2018). Recent studies suggested that foliar Se application at later growth stages is more effective for increasing the Se content of plant (Deng et al., 2017; Dinh et al., 2019; Wang et al., 2020). Nevertheless, the systematic study on the accumulation of Se in wheat grains with different Se application methods is still lacking.

No specific Se uptake pathways in plants are found yet since Se is not an essential element for plants (Dinh et al., 2019). Meanwhile, due to similar chemical properties between Se and sulfur (S), the uptake of Se(VI) occurs along the same pathway as sulphate, which occurs mainly through *SULTR1;1* and *SULTR1;2* transporters using an active transport process (Izydorczyk et al., 2020). Se(IV) is taken up by roots as HSeO_3_^–^ by the members of phosphate transporter *Pht1* family using aquaporins (Zhang et al., 2014; White, 2018).

Wheat is one of the staple crops for more than one third of the world’s population (Boldrin et al., 2018), and is the most efficient Se accumulator among the common cereals (Poblaciones et al., 2014; Dinh et al., 2018). Nevertheless, 63% of the wheat grown in China, for example, is deficient in Se with an average concentration at 64.6 μg Se kg^–1^, which provides insufficient daily Se for sustaining human health (Liang et al., 2020). Thus, agronomic Se biofortification of wheat may be one of the best approaches to increase Se intake by human. Studies showed that most Se absorbed by wheat was distributed in the grains (Keskinen et al., 2010; Eiche et al., 2015; Wang et al., 2017). Past research efforts generally focused on Se content in root, grain, leaves, stem, and glume of wheat (Lyons et al., 2005; Shinmachi et al., 2010; Eiche et al., 2015; Nawaz et al., 2015; Liu et al., 2016; Wang et al., 2020). Importantly, the rachis and nodes may also play important roles in transporting Se from leaves and roots to the developing grains in panicles and transferring Se from the xylem to phloem, respectively (Chen et al., 2018). However, there is no systematic study on the effects of different Se application methods on Se accumulation in various parts of wheat, especially in nodes and rachis.

Selenomethionine (SeMet) is the primary Se species in wheat grains (Poblaciones et al., 2014; Eiche et al., 2015; Wang et al., 2020). Lu et al. (2018) also reported that in Se-enriched wheat, SeMet accounted for 44.2% of the total Se, while selenocystine (SeCys_2_) and methylselenocysteine (MeSeCys) accounted for 2.6 and 0.3% of the total Se, respectively (Carey et al., 2012). In general, most exogenous Se was accumulated in wheat leaves after foliar Se application (Wang et al., 2020), however, some research questions remain. For example, is there a correlation between Se speciation in leaves and grain of wheat? Is the Se speciation in these tissues affected by different Se application methods, rates, species, and growth stages of wheat?

The production of Se-enriched wheat can be an important step in eliminating the negative impact of Se deficiencies in low Se areas. In this regard, bioaccessibility of Se from edible wheat tissues is important to understand. The bioaccessibility of Se using in vitro simulated gastrointestinal digestion test (PBET) refers to the portion of a nutrient, e.g., Se in a food product, that can be found dissolved in gastric (G) and intestinal (I) phases, and be potentially absorbed and utilized by organisms (Zhou F. et al., 2021). The order of bioaccessibility of Se for different gastrointestinal digestion simulation methods in gastricand intestinal was as follows: PBET > UBM (unified bioaccessibility method) > SBRC (solubility bioaccessibility research consortium method) > IVG (in vitro gastrointestinal method; Zhou et al., 2020). The PBET method has become a common evaluation method for evaluating the bioaccessibility of Se (Zhou et al., 2019; Muleya et al., 2021). Hitherto many studies on Se bioaccessibility in green vegetables have been carried out, including on Se-enriched leeks (Lavu et al., 2012), potato (Dong et al., 2021), lettuce (Do et al., 2017), and Se-enriched crops, such as maize (Muleya et al., 2021). For example, Muleya et al. (2021) found that the mean bioaccessibility of Se was 73.9 ± 8.5% with no significant difference across all selected crops (maize, groundnut, and cowpea). Especially, Lu et al. (2018) showed that the bioaccessibility of Se in Se-enriched wheat and soybeans was 90%, corn and broccoli was 80%, and cardamine was 50%. In wheat, however, the embryo and endosperm are the main storage sites of Se in wheat grain, about 80–90% of Se is stored in wheat flour after grinding the grains (Lyons et al., 2005), and nearly 5% of the whole grain Se was lost in the milling process (Govasmark et al., 2010). To date, it has not yet been reported whether the Se bioaccessibility in whole wheat and in white all-purpose flours is significant different, and whether Se application methods affect the Se bioaccessibility.

Currently, the main methods used to explore the uptake, translocation, and transformation of Se in crops can be divided as: hydroponic experiment, pot experiment, and field experiment (Wang et al., 2019, 2020, 2021; Xia et al., 2020). The environment for hydroponic experiments is quite different from the actual soil environment, which is completely different from field experiments (Wang et al., 2019). It is difficult to analyze the environmental process and influencing factors of field experiments, since the conditions of field experiments are not well controlled, and temperature and humidity will affect the experimental results (Wang et al., 2020; Xia et al., 2020). Pot experiment can both study the mechanism and be closer to the actual soil environment (Wang et al., 2021). Given above, the pot experiment is more suitable at present and can accurately explore the reality. Wheat, as a world-wide consumed crop that has a strong Se accumulation ability (Wang et al., 2021), was selected as the research crop in this study. We hypothesized that different Se application treatments will affect the growth of wheat and then influence the Se speciation and Se bioaccessibility in Se-enriched wheat flours. The main objectives of this study were as follows: (1) compare the effects of different Se application methods on the growth and Se accumulation in different parts of wheat; (2) explore the influences of two Se application methods on the Se speciation in the leaves and grains of wheat; and (3) ascertain the differences of Se bioaccessibility under different Se treatments in whole wheat and white all-purpose flours.

## Materials and methods

### Materials and reagents

The pot experiment was carried out in a greenhouse at Northwest Agriculture and Forestry University in Yangling, Shaanxi from year 2018 to 2019. Tested soil was collected from the non-polluted farmland around Northwest A&F University, which has never received applied exogenous Se. After air-drying, homogenizing, and grinding, the soil was passed through 2 and 0.149 mm sieve for physical and chemical analysis determined according to Bao (2000). The relevant physicochemical properties are as follows: soil pH, 8.14; carbonate content, 118.0 g kg^–1^; organic carbon, 8.53 g kg^–1^; cation exchange capacity, 23.34 cmol(+) kg^–1^; amorphous aluminum, 0.40 g kg^–1^; amorphous iron, 1.20 g kg^–1^; clay, 39.6%; and total Se, 0.139 mg kg^–1^.

Winter wheat seeds (*Triticum aestivum* L, Xiaoyan-22) were provided by a commercial seed company of Northwest Agriculture and Forestry University. Wheat seeds with full grains were selected for consistent size and no pest infestation damage, then disinfected with 5% (V/V) H_2_O_2_ for 30 min and washed thrice with deionized water.

Se(IV) was sodium selenite (Na_2_SeO_3_, ≥ 97%; Tianjin Fuchen Chemical Reagent Factory), and Se(VI) was sodium selenate (Na_2_SeO_4_, ≥ 98%; Beijing Xiya Chemical Industry Co., Ltd), both were analytical pure reagents. The organic Se (SeMet, SeCys_2_, and MeSeCys) were all purchased from Sigma-Aldrich company and used for the determination of Se speciation in grain and leaves of wheat. Pepsin, sodium malate, sodium citrate, lactic acid, acetic acid, bile salt and trypsin, which were used for the determination of Se bioaccessibility, were all purchased from Yuanye Biological Technology Co., Ltd.

### Experimental design

A complete block design was used in this study, two species of exogenous Se (Se(IV) and Se(VI)), three application rates of Se (0.5, 1, and 2 mg kg^–1^) were selected in soil Se application. For foliar Se treatments, two species of exogenous Se (Se(IV) and Se(VI)), three application rates of Se (5, 10, and 20 mg L^–1^) were applied at two growth stages of pre-flowering stage (F1) and pre-filling stage (F2). A total of 19 treatments were used in this experiment and each treatment was replicated three times.

Pots had an inner diameter of 32 cm and a depth of 20 cm, and were filled with 8 kg soil. All soil samples were completely air-dried, ground, and prepared through a 5 mm sieve. Se(VI) and Se(IV) solutions were prepared according to the designed Se application rates and then evenly sprayed into the soil and mixed. The soil moisture was adjusted to 70% of the water holding capacity. After full mixing, the sprayed soil was allowed to equilibrate at 25°C for 30 days (Li et al., 2016), and deionized water was added every 2–3 days during the equilibrium stage.

During the sowing period, 0.15 g N (urea, analytical pure) and 0.033 g P (potassium dihydrogen phosphate, analytical pure) were applied to each kilogram of soil, and 0.15 g kg^–1^ nitrogen fertilizer was applied at regreening stage of wheat. 20 seeds were sowed into each pot. Two weeks after the emergence of seedlings, the seedlings were thinned to 10 plants per pot. The pots were weighed and watered every 4–14 days during the wheat growing season. For the foliar Se application, the Se solution was sprayed evenly on the plants during the pre-flowering stage (April 2019) and the pre-filling stage (May 2019) of growth. Specifically, 100 mL Se (IV) or Se (VI) solution (5, 10, and 20 mg L^–1^) were mixed into water with 0.1% surfactant. Foliar Se was applied three times (100 mL each time, respectively) in intervals of 5 days to ensure that Se was fully absorbed by wheat leaves. Each pot was sprayed with a total of 1.5, 3, and 6 mg Se(IV) and Se(VI), respectively. Moreover, during the foliar application process, the soil surface was covered with plastic film to avoid Se from dripping onto the soil.

### Sample collection

The height and length of rachis and the effective ear number of wheat were measured after wheat harvest (June 2019). The harvested wheat was first washed with tap water thrice to remove dust and other impurities, rinsed with deionized water thrice, and then dried with absorbent paper. Meanwhile, each wheat plant was divided into nine parts: root, stem, leaf, glume, grain, sheath, first internode, first node, and rachis (Chen et al., 2018). After weighing fresh weight (FW) of roots, each replicate was placed into paper bags, dried at 90°C for 30 min and at 55°C for 3 days, and then dry weight (DW) was recorded. All parts of wheat were ground into powder to determine the total Se content. In addition, fresh grain and leaf tissue samples were freeze-dried, grounded, and then stored at 4°C for the determination of Se speciation (described later). Flour and bran were separated by a sieve (0.149 mm), weighed, mixed to obtain whole wheat and white all-purpose flours, and ground into powder for the determination of Se bioaccessibility (see section “*In vitro* simulated gastrointestinal digestion test”).

### Determination of samples

#### Selenium content in various parts of wheat

The total Se content was determined via hydride atomic fluorescence spectrometry (AFS, Beijing Jitian AFS-930 dual-channel atom fluorescence photometer, Beijing, China) after wet-acid digestion. The specific procedure has been described by Wang et al. (2020).

#### Selenium speciation in wheat grains and leaves

Selenium speciation was determined by HPLC-ICP-MS. First, 0.2000 g freeze-dried grains or leaves was taken into a centrifuge tube, 20 mg protease XIV and 5 mL water were added, vortexed for 30 s, ultrasonic extraction for 3 h in a 37°C water bath and shook several times during the period. Second, the sample was centrifuged at 9,000 r min^–1^ for 10 min at 4°C. The supernatant was collected after pouring through a 0.22 μm filter membrane, and then analyzed using the HPLC-ICP-MS system. The instrument conditions are as follows: for the HPLC; Hamilton PRP-X100 anion exchange column (250 mm × 4.1 mm, 10 μm) was used, the column temperature was room temperature, the mobile phase was 40 mmol L^–1^ diammonium hydrogen phosphate (pH = 6.0 adjusted with 10% formic acid) at a flow rate of 1.0 mL min^–1^, and the injection volume was 100 μL. For the ICP-MS; RF power was 1,550 W, RF matching voltage was 1.8 V, sampling depth was 8 mm, atomization chamber temperature was 2°C, plasma gas flow rate was 15.0 L min^–1^, the flow rate of carrier gas was 0.65 L min^–1^, the mode was high He collision mode, the flow rate of collision gas was 4.5 mL min^–1^, peristaltic pump speed was 0.3 r s^–1^. The detection mass number m/z = ^78^(Se), and the integration time was 0.5 s. At the same time, Se-enriched yeast of SELM-1 was used as the quality control sample, the measured content of SeMet in the quality control sample was 3,236 ± 21 mg kg^–1^, the standard value was 3,389 ± 173 mg kg^–1^, the recovery rate was 95.5%.

#### *In vitro* simulated gastrointestinal digestion test

According to the method of Zhou et al. (2019), the PBET method was carried out, and divided into two stages: gastric (G) digestion and intestinal (I) digestion. The specific steps are as follows:

(1) G: 1.000 g sample was accurately weighed into 100 mL polyethylene centrifuge tube, and 50 ml fresh gastric juice (pH 2.5) were added into a constant temperature (37°C) water bath for digestion at 150 rpm for 1 h. The obtained digestive juice was centrifuged at 4,000 rpm for 10 min and 10% of the supernatant was removed and stored at 4°C for Se content determination.
(2) I: the pH of the remaining digestive juice was adjusted to 7.0 by 10% (m/v) NaOH. Then 5 mL intestinal fluid were added and digested in a constant temperature water bath (150 rpm, 37°C) for 4 h. The obtained digestive fluid was centrifugated at 4,000 rpm for 10 min, and the supernatant was stored at 4°C for further Se content determination.

Moreover, the total Se content in G and I sample (2 mL of digestive fluid) were determined by the method already described in section “Selenium content in various parts of wheat.” The composition of gastric juice and intestinal juice was the same as Zhou et al. (2019).

### Statistical analysis

Pearson correlation analysis and variance analysis were performed by SPSS 20.0 (IBM, United States; Duncan method was used for significance test at α = 0.05). The data in the chart are the averages of three replicates, and the data were calculated using the following Eqs.


(1) $${\text{TF}}_{\text{a}-\text{b}}=\frac{{\text{C}}_{\text{b}}}{{\text{C}}_{\text{a}}}$$

where TF*_*a*_*_–_*_*b*_* represents the Se translocation factor from part “b” to part “a” of wheat (Dinh et al., 2019). *C*_*a*_ and *C*_*b*_ represent the Se content in part “a” or part “b” of wheat, respectively (μg g^–1^). “a” or part “b” refer to different parts of “root,” “first node,” “rachis,” “grain,” and “leaves.”


(2) $$\mathrm{BA}\%=\frac{\mathrm{Se}\mathrm{in}G/I}{\mathrm{Se}\mathrm{in}\mathrm{sample}}\times 100\%$$

where BA% represents the Se bioaccessibility of whole wheat and white all-purpose flours. Selenium in G/I was the Se content in gastric or intestinal phase of the sample, mg kg^–1^. Se in sample indicates the Se content in the corresponding sample, mg kg^–1^.


(3) $$\mathrm{LS}\%=\frac{\mathrm{lost}\mathrm{Se}\mathrm{content}}{\mathrm{Se}\mathrm{in}\mathrm{sample}}\times 100\%$$

where LS% represents the Se lost proportion with different flour yield. The lost Se content (μg g^–1^) is the difference between the Se content of whole wheat and white all-purpose flours with different Se treatments.

## Results

### Basic growth index of wheat

**FIGURE 1:**
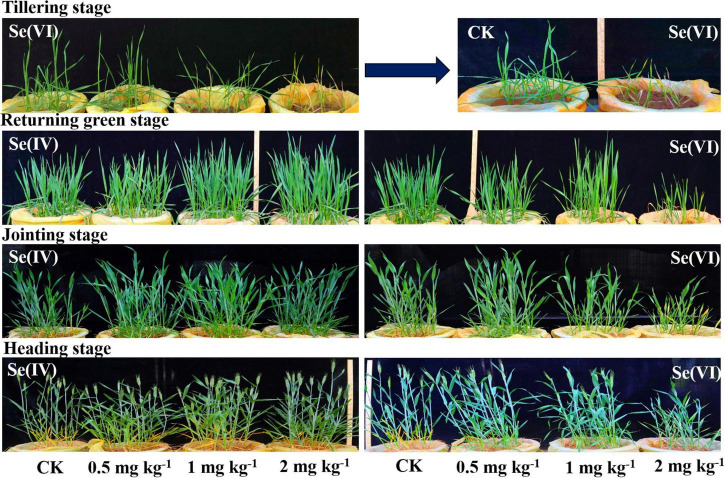
The growth of wheat at different growth stages under different Se treatments. Se(IV) refers to selenite treatment and Se(VI) refers to selenate treatment.

Figure 1 shows the growth of wheat at different growing stages. Albino seedlings appeared at tillering stage when 2 mg kg^–1^ Se(VI) was soil applied, indicating that the 2 mg kg^–1^ Se treatment has little inhibition on wheat growth. However, the growth of wheat appeared to slow down due to the biological dilution effect at the later growth stage of wheat.

Supplementary Table 1 illustrated that different Se treatments had significant (*p* < 0.05) effects on the biomass and grain yield of wheat. Soil application with 2 mg kg^–1^ Se(IV) resulted in the highest grain yield of wheat, which was about 6% higher than control treatment. Compared with control, all soil Se application treatments reduced the yield of wheat (by 4–5% by Se(IV) treatments, except at 2 mg kg^–1^ Se(IV) (yield increased by 10% compared with 0.5 mg kg^–1^ treatments), and 4–62% in Se(VI) treatments). However, no significant effects (*p* > 0.05) were observed in the grain yield of wheat at different foliar Se application treatments, irrespective of the Se species, application rates, and application stages.

We note that soil Se(IV) application treatments significantly (*p* < 0.05) increased the biomass of wheat (7–11%), compared with control, while the application of Se(VI) increased Se application rates both significantly (*p* < 0.05) reduced wheat biomass (2–59%). Compared with 2 mg kg^–1^ Se(VI) treatment, the biomass of wheat treated with 0.5 mg kg^–1^ Se(VI) increased by 58%. No significant (*p* > 0.05) effects on the biomass of wheat were found among different foliar Se application treatments, irrespective of exogenous Se species, application rates, and application stages.

### Selenium content in wheat grain

**FIGURE 2:**
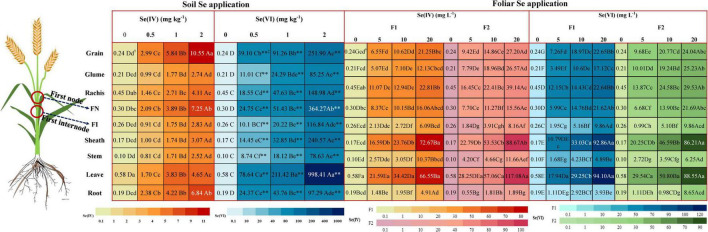
The Se content in different parts of wheat under different soil and foliar Se treatments. F1 represents pre-flowering stage and F2 represents pre-filling stage. Different lowercase letters of “a”–“h” indicate significant (*p* < 0.05) differences between different parts of wheat at each treatment. Different capital letters of “A”–“G” indicate significant (*p* < 0.05) differences between different rates of soil Se(IV), soil Se(VI), foliar Se(IV), and foliar Se(VI) application on Se concentration in the same parts of wheat (*p* < 0.05). “^**^” and “*” indicate the significant (*p* < 0.01; *p* < 0.05) differences between the same part of wheat and the same Se application rates of different Se treatments, respectively.

The harvested wheat plants were divided into nine parts: root, stem, leave, sheath, first internode, first node, rachis, grain, and glume (Figure 2). We observed that application of Se, either via foliar or soil methods, significantly (*p* < 0.05) increased the Se content in each part of wheat in comparison to control. The Se content increased with higher rate of Se application. A significant (*p* < 0.05) increase of 72 and 84% of the Se content in grains of wheat was observed at soil application 2 mg kg^–1^ Se(IV) and Se(VI) treatments, compared with 0.5 mg kg^–1^ treatment of either form. Meanwhile, the Se content in grains with foliar application rate of 20 mg L^–1^ Se(IV) and Se(VI) increased remarkably (*p* < 0.05) by 68–69% and 60–68% at pre-flowering and at pre-filling stages, respectively, compared with the corresponding 5 mg L^–1^ Se application treatments.

Regardless of application method of Se(IV) or Se(VI), the Se content of the first node of wheat was always higher than that of the first internode. Meanwhile, irrespective of the Se application rate and method, Se(VI) treatments significantly (*p* < 0.05) increased the Se content in each part of the wheat (90–99.5%), compared with Se(IV) treatments (except foliar application of 20 mg L^–1^ Se(VI) applied at pre-filling stage). Compared with foliar Se(IV) treatment, foliar application of Se(VI) at pre-flowering stage and pre-filling stage significantly (*p* < 0.05) increased the Se content of wheat grains by 6–44% and 3–28%, respectively. In addition, the Se content of wheat grains from foliar Se(IV) and Se(VI) application at pre-filling stage significantly (*p* < 0.05) increased by 22–30% and 6–25% than that applied at pre-flowering stage, respectively.

Foliar Se(IV) application significantly (*p* < 0.05) increased the Se content of wheat grains compared with corresponding soil application treatments, irrespective of the application stages. Specifically, the Se content of wheat grains sprayed with Se(IV) significantly (*p* < 0.05) increased by 45–54% and 61–68% at pre-flowering and pre-filling stages, respectively, compared with soil Se(IV) application. In contrast to foliar Se(IV) treatment, soil application of Se(VI) significantly (*p* < 0.05) increased the Se content of wheat grains compared with its foliar application. The Se content of wheat grains in the soil application treatments was significantly (*p* < 0.05) increased by 79–91% and 75–90% at the pre-flowering and pre-filling stages, compared with foliar application treatments, respectively.

### Translocation factor of Se in wheat plant

**FIGURE 3:**
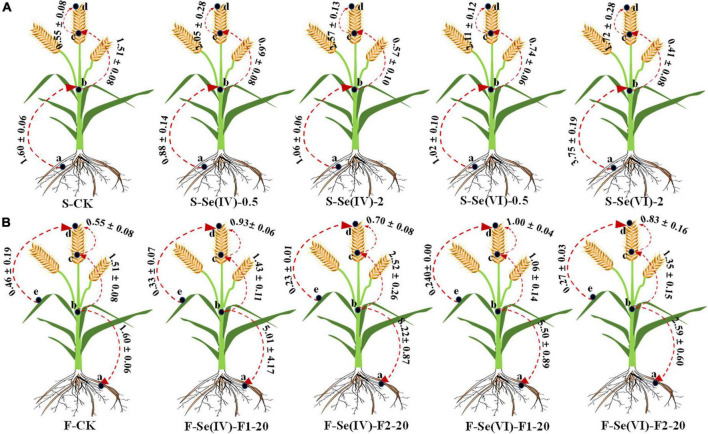
The TF values of wheat under different soil and foliar Se treatments. CK = control, F1 represents pre-flowering stage and F2 represents pre-filling stage; **(A)** represents TF of wheat with soil Se application, **(B)** represents TF of wheat with foliar Se application, “a,” “b,” “c,” “d,” and “e” denote different parts of “root,” “first node,” “rachis,” “grain,” and “leaves,” respectively. The direction of the arrow indicates the direction of TF.

Translocation factor (TF) can be used to reflect the translocation capacity of plant from source to sink (Dinh et al., 2019). Figure 3 showed the effects of different Se treatments on TF among different parts of wheat. According to the different Se application methods (soil and foliar Se application), the TF of Se in wheat was divided into two parts: (a) soil Se application: TF_first nodes/root_, TF_rachis/first nodes_, and TF_grains/rachis_, (b) foliar Se application: TF_root/first nodes_, TF_rachis/first nodes_, TF_grains/rachis_, and TF_grains/leaves_.

Compared with control, soil Se application treatments significantly increased the TF_grain/rachis_ of wheat (1.2–2.1), while reduced the TF_rachis/first nodes_ (0.6–1.1) and TF_first nodes/root_ (0.4–0.7; except at the soil application of 2 mg kg^–1^ Se(VI)). Moreover, when Se(IV) was soil applied, the TF_grains/rachis_ increased with the higher application rate of exogenous Se, and the TF_grains/rachis_ decreased when Se(VI) was soil applied. Although soil Se application reduced the TF_first nodes/root_, TF_first nodes/root_ increased with a higher rate of exogenous soil Se applied, irrespective of the Se species. Regardless of the application stages, rates, and species of Se, foliar spraying of exogenous Se had no significant (*p* > 0.05) effects on TF_grains/leaves_. Compared with control, foliar Se application of both forms of Se increased the TF_grains/rachis_ (0.1–0.8) in wheat. Compared with pre-filling stage, the TF_grains/rachis_ in wheat increased at pre-flowering stage ((Se(IV): 0.2–0.5; Se(VI): 0.1–0.9)). In addition, spraying Se(IV) at pre-filling stage significantly (*p* < 0.05) increased the TF_rachis/first nodes_ (0.8–1.1) and the TF_root/first nodes_ (0.9–8.4), compared with control.

### Selenium speciation in grains and leaves of wheat

#### Chromatogram of wheat grains and leaves

**FIGURE 4:**
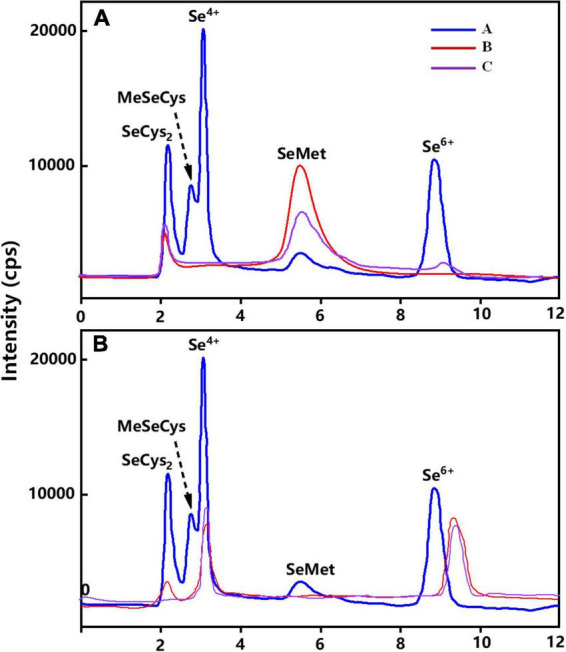
Chromatogram of Se species in wheat grain and leaves under different soil and foliar Se treatments. **(A)** represents the Se species in wheat grain and **(B)** represents the Se species in wheat leaves. “A” represents Se species in wheat grain in standard compounds, “B” represents Se species in wheat grain under soil application of Se(IV), and “C” represents Se species in wheat grain under soil application of Se(VI).

**FIGURE 5:**
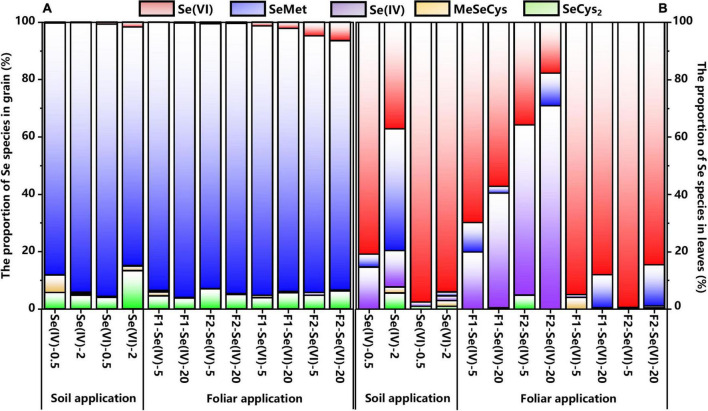
The proportion of Se species in wheat grain and leaves under different soil and foliar Se treatments. **(A)** represents the Se proportion in wheat grain and **(B)** represents the Se proportion in wheat leaves.

Figure 4A showed the percentages of Se species or Se compounds identified in wheat grains under different Se treatments. Irrespective of the Se application methods, Se speciation in wheat grains treated with different forms of exogenous Se were mainly organic Se (93–100%). Organic Se was mainly composed of SeMet (87–96%) and SeCys_2_ (4–13%) (Figure 5), while Se(VI) was the main inorganic Se species in wheat grains (1–6%). Figure 4B represents the chromatogram of wheat leaves, it can be seen that Se(VI) was the main Se species in wheat leaves.

#### Distribution of Se speciation in grains of wheat

No significant differences were observed for the percentages of SeMet in wheat grains among soil (83–95%) and foliar (87–96%) Se application treatments, while the percentages of SeMet varied with Se application rate (Figure 5). The percentage of SeMet in soil Se(IV) and Se(VI) treatments at 0.5 mg kg^–1^ increased by 6% and decreased by 12%, respectively, compared with the 2 mg kg^–1^ treatment, respectively. However, there was no significant (*p* > 0.05) differences among the foliar Se application rates.

The species of exogenous Se also affected the percentages of SeMet in grains. With soil application rate at 0.5 mg kg^–1^, the percentage of SeMet in wheat grains increased by 7% in Se(VI) treatment compared with Se(IV) treatment, but decreased by 11% at 2 mg kg^–1^ Se(VI) soil treatment. However, the percentage of SeCys_2_ in wheat grains increased by 9% with 2 mg kg^–1^ soil Se(VI) treatment compared with 0.5 mg kg^–1^ Se(VI) soil treatment. Moreover, foliar Se(IV) application increased the percentage of SeMet in wheat grains, compared with Se(VI) application. We found that compared with Se(VI) treatments, the percentage of SeMet in wheat grains treated with Se(IV) increased by 3–7%. The percentage of SeMet measured in wheat grains produced from foliar Se treatment with high rate of Se(VI) at pre-flowering and pre-filling stages, increased by 9 and 5%, respectively, compared with soil Se treatments.

#### Distribution of Se speciation in leaves of wheat

Regardless of the application methods, inorganic Se (50–100%) was the major Se species in wheat leaves in all the treatments, with Se(IV) and Se(VI) accounting for 1–71% and 18–99% of total Se, respectively, while SeMet (1–42%) was the main organic Se species (Figure 4B). Moreover, Se(IV; 20–71%) and Se(VI; 85–99%) were the main Se species in wheat leaves when treated with Se(IV) or Se(VI) application via foliar or soil Se application, respectively. In addition, the percentage of Se(IV) was reduced by 31–39% after foliar Se(IV) application at pre-flowering stage, but the percentage of Se(VI) was increased by 34–39%, compared with pre-filling stage. Compared with Se(IV) treatments, the perecentage of SeMet increased by 12% and 14% in wheat leaves applied with Se(VI) at pre-flowering stage and pre-filling stages, respectively. The percentages of SeMet were increased at pre-filling stage compared with pre-flowering stage. Meanwhile, a 10% and 3% increase was observed in leaves at 20 mg L^–1^ Se(VI) treatment at pre-flowering stage and pre-filling stage, respectively, compared with foliar Se(IV) application.

Irrespective of the application methods of Se, the percentage of organic Se in wheat leaves with soil application at 2 mg kg^–1^ Se(IV) rate was the highest, which was 35–49% higher than other treatments. Except for soil Se(IV) application at 2 mg kg^–1^, the percentage of organic Se in wheat leaves sprayed with 20 mg L^–1^ Se(VI) increased by 8% and 11% at pre-flowering and pre-filling stages, respectively. The percentage of SeMet in wheat leaves with soil Se(IV) application at 2 mg kg^–1^ increased by 40% compared with the corresponding foliar application treatments at pre-flowering stage. The percentage of Se(VI) in wheat leaves with soil application at 20 mg L^–1^ Se(VI) increased by 8% and 9% applied at pre-flowering stage and pre-filling stages, respectively, compared with foliar application treatments.

### Selenium bioaccessibility in wheat flour

#### Selenium lost in whole wheat and white all-purpose flour

The whole wheat (100% wheat) and white all-purpose flour (70% wheat flour and 30% bran) were obtained by controlling different proportions of flour and bran of wheat. Selenium lost was calculated as the difference between the Se content of whole wheat and white all-purpose flour. Irrespective of the Se application methods, the percentage of Se lost in wheat flour process among the different Se treatments ranged from 12 to 68%. For soil Se application at 2 mg kg^–1^ Se(IV) and Se(VI) treatments, the percentages of Se lost in wheat flour were 4% and 8%, respectively, compared with the 0.5 mg kg^–1^ Se(IV) and Se(VI) treatments. The percentages of Se lost in wheat flour produced from plants sprayed with 5 mg L^–1^ Se(VI) increased by 40% (pre-flowering stage) and 23% (pre-filling stage), compared with 20 mg L^–1^ Se(VI) treatment.

In general, flour produced from foliar Se application had a higher percentage of Se lost in flour produced from soil Se application. The percentage of Se lost in wheat flour treated with foliar Se(IV) application was 2–12% (pre-flowering stage) and 43–51% (pre-filling stage) higher than that of the soil Se treatments (except for spraying Se(VI) at pre-filling stage). When Se(VI) was sprayed, the percentage of Se lost in wheat flour was 28% (at pre-filling stage) and 42% (at pre-filling stage) higher than the soil Se(VI) treatment.

#### The bioaccessibility of Se in wheat flour

The bioaccessibility of Se in whole wheat and white all-purpose flours are shown in Figure 5 for different Se treatments. The bioaccessibility of Se in white all-purpose flour was higher than that in whole wheat flour. In the gastric stage (G), the bioaccessibility of Se was 6–27% and 6–34% in whole wheat and white all-purpose flour, respectively. Meanwhile, the bioaccessibility of Se in whole wheat flour was 9–34% and 10–38% in white all-purpose flours in the intestinal phase (I).

Irrespective of the Se application methods and Se species, the Se bioaccessibility in wheat flour (either whole wheat and white all-purpose flours) produced from soil at 2 mg kg^–1^ increased by 6–13% compared with 0.5 mg kg^–1^ treatments, except for the soil application of 2 mg kg^–1^ Se(VI) treatments. Compared with the 0.5 mg kg^–1^ Se(VI) treatment, the Se bioaccessibility in whole wheat and white all-purpose flours decreased by 13% (G) and 16% (I), and 15% (G) and 17% (I) in soil at 2 mg kg^–1^ Se(VI) treatment, respectively. In addition, the Se bioaccessibility in Se(VI) treatments in both foliar and soil Se application was higher than that in Se (IV) treatment, except soil application at 2 mg kg^–1^ Se(VI). In soil Se application, the bioaccessibility of Se in wheat flour of Se(VI) treatment increased by 4% in both G and I (in whole wheat flour), compared with Se(IV) treatment. Compared with foliar Se application at pre-flowering stage, foliar Se application at pre-filling stage increased the bioaccessibility of Se in whole wheat (3–4%) and white all-purpose flours (2–8%) in G and I (1–3%, whole wheat and 1–6%, white all-purpose flour).

## Discussion

### Effects of selenium application methods on the growth of wheat

Selenium has been reported to be a beneficial element that can promote plant growth and improve plant resistance to stress although the essentiality of Se to plants is still questionable (Schiavon et al., 2015). Others have reported that the excessive accumulation of Se in plants may also inhibit the growth of crops (Wang et al., 2019). In this study, we found that soil application at 2 mg kg^–1^ Se(VI) significantly reduced plant height, effective ear number, and rachis length of wheat compared with 0.5 mg kg^–1^ Se (VI) treatment (Figure 1, Supplementary Figures 1, 3), indicating that 2 mg kg^–1^ Se(VI) has a certain toxic effect on wheat growth. Based on this observation, it appears that 1 mg kg^–1^ can be used as the tolerance limit of Se(VI) in a wheat Se biofortification strategy. The grain yield of wheat in 0.5 mg kg^–1^ Se(VI) treatments increased by 61% compared with 2 mg kg^–1^ Se(VI) treatments. Similarly, this study also found that the highest grain yield was significantly (*p* < 0.05) obtained in soil applied with 2 mg kg^–1^ of Se(IV), which was about 6% higher than the control treatment (Supplementary Table 1).

Previous studies have obtained varied results about different Se application methods. For example, Lara et al. (2019) and Ducsay et al. (2016) found that foliar Se application increased the yield of wheat. A two-year field study on the purple-grained wheat and common wheat showed that the soil Se application increased shoot dry weight and grain yield, while there was no significant (*p* > 0.05) difference between foliar Se application and control treatment. Zhang and Zhou (2019) found that neither foliar nor soil Se application had significant effects on rice yield and biomass (*p* > 0.05). However, in soil Se application, compared with the selenite application (1 mg kg^–1^), the grain and the biomass yield of ZM-9023 significantly (*p* < 0.05) increased by about 15% for selenate application (10 mg kg^–1^; Wang et al., 2021). The discrepancy in results may be attributed to the different growth stages and methods of Se application. Although soil Se application during the sowing period didn’t affect the uptake efficiency of Se immediately (Curtin et al., 2006), Se can play a role in the entire growth cycle of wheat. Wheat can only absorb exogenous Se from pre-flowering stage or pre-filling stage to maturity stage in foliar Se treatments. Although foliar Se application in a wheat Se biofortification strategy is more efficient than soil Se application for increasing Se concentration in wheat, it has no significant effect on wheat yield.

The reason why application 0.5 mg kg^–1^ selenite increased yield may due to that soil Se application may influence the soil microorganisms and thereby promote the growth, development, and yield of wheat (the entire growth stage; Dinh et al., 2019). In addition, the increase of crop yield by exogenous Se application may be related to the improvement of crop’s antioxidant capacity (D’Amato et al., 2018). Studies showed that applying appropriate rates of exogenous Se increased the antioxidant capacity of crops (Gupta and Gupta, 2017). The activities of SOD, POD, CAT, and other enzymes all increased with the application of exogenous Se is the main reason for the increased yields reported (Nawaz et al., 2015). However, high Se rate application can also be toxic to crops reduce their antioxidant capacity and yields (as we observed on decreased yield with high rate of Se (VI)). Therefore, application of appropriate rates of Se may reduce the oxidative stress and increase the biomass and yield of wheat. The underlying mechanisms of the increase in yield still need to be further studied.

### Effects of selenium application on selenium uptake and translocation in wheat

Selenium content in wheat grains was higher with either soil or foliar Se application compared with control (Figure 2), which is consistent with the results of Keskinen et al. (2010) and Wang et al. (2019). They all found that most of the Se absorbed by wheat was distributed in the grain, indicating Se application can improve the Se content in grain. In this study, we separated wheat into nine parts (sheath, first internode, first node, and rachis haven’t been systematically studied) for the first time. Consistent with previous studies (Nawaz et al., 2015; Boldrin et al., 2018), this study observed that soil application of Se(VI) significantly (*p* < 0.05) increased the Se content in each part of wheat (90–99.5%), and spraying Se(VI) increased the Se content of wheat grains (3–44%) compared with Se(IV) treatment. This increase in Se accumulation with selenate may be attributed to the different transport mechanism of Se(VI) and Se(IV) in plants. The uptake and translocation of these two inorganic forms of Se by plants is an energy-consuming process (Li et al., 2008). Due to the similar chemical properties between Se(VI) and sulfate, Se(VI) enters the roots of plants through the sulfate transport system (Shinmachi et al., 2010). Se(VI) absorbed by plants is easily transported from roots to shoots with no speciation change, it is reduced to Se(IV) in leaves, and then converted into organic Se compounds, which are then distributed to other plant tissues (Gupta and Gupta, 2017; Wang et al., 2020). However, Se(IV) is more easily converted into organic forms (including SeMet and its oxide, SeOMet) after being absorbed by plant roots and mainly accumulate in root, only a small part can be transported to shoots (Li et al., 2008).

Rachis is the organ connecting the stem and grain of wheat (Chen et al., 2018). Selenium applied by fertilizers is absorbed by leaves (foliar applied) and roots (soil applied) of wheat and are eventually transported to the developing grains through the rachis of wheat. In this study, the Se content in the rachis and grains of wheat were higher than other parts of the plant for all treatments with foliar Se application (Figure 2). In this regard, recent studies suggested three pathways of foliar Se uptake, including cuticular, plant stomata, and trichomes (Zhou J. et al., 2021). Foliar application conditions can also affect the absorption of Se fertilizers (Shahid et al., 2017), and the leaf physical characteristics such as stomatal density, roughness, and epidermal wax layer, may affect the deposition of fertilizers on the surface of leaves (Chen et al., 2018). Compared with soil Se(IV) application, the grains of wheat treated with foliar application of Se(IV) have a higher Se content (Figure 2), indicating that foliar Se application can efficiently increase the translocation of Se to the grain, especially in the phloem (Adrees et al., 2015). The Se content of wheat is determined by the transport of xylem-mediated Se transport from the root to the aerial part and phloem-mediated Se (Kato et al., 2010).

This study found that irrespective of the Se application methods, the Se content in the first node was higher than that of the first internode. Except for wheat grains and leaves, the Se content in first internodes and nodes was relatively high, which was consistent with findings reported by Zhou J. et al. (2021). These results showed that the first node plays an important role in the storage of exogenous Se in wheat. Although no studies have explored the effect of application of exogenous Se on the gene expression in first nodes, it was speculated that the upregulation of transporter-related genes helped allocate the transfer of Se to grain. Therefore, further research on gene expression in nodes after Se applications should be explored.

Selenium applied by foliar application can enter the foliage through the epidermis or stomata, and then transported to the edible parts of plant (Luo et al., 2021). However, this study found that Se mainly remained in the leaves and sheaths after foliar Se application (Figure 2), although the Se content of wheat grains was significantly increased by 22–30% for Se application at pre-filling stage compared to pre-flowering stage. Further comparison of the TF of Se in different parts of wheat showed that the TF_*rachis/first nodes*_ increased when exogenous Se was applied at pre-flowering stage compared with pre-filling stage (Figure 3). This result indicates that spraying exogenous Se at pre-filling stage increased the transfer of Se from the nodes to rachis, which shows that the efficiency of foliar Se application is higher at pre-filling stage. This observation is consistent with results obtained from field trials with wheat of Deng et al. (2017) and Wang et al. (2019).

### Effects of exogenous selenium application on either selenium species distribution or selenium speciation variation

**FIGURE 6:**
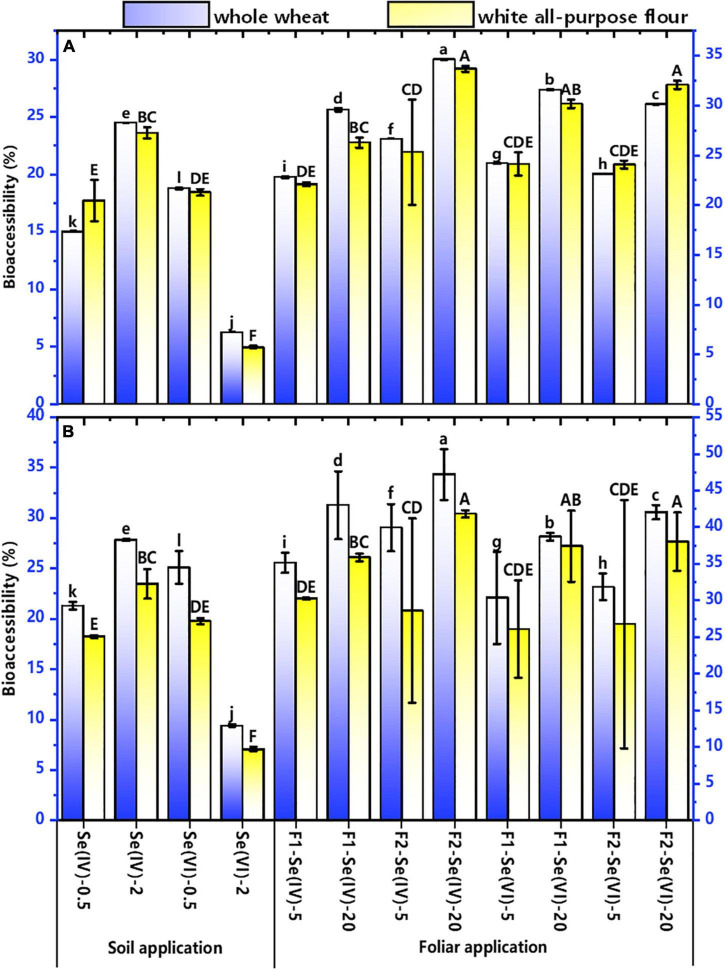
The bioaccessibility of Se in whole wheat and white all-purpose flour under different soil and foliar Se treatments. The bioaccessibility of Se in whole wheat and white all-purpose flour under different soil and foliar Se treatments. **(A)** represents the bioaccessibility of Se in gastric phase of whole wheat and white all-purpose flour with different Se treatments; **(B)** represents the bioaccessibility of Se in intestinal phase of whole wheat and white all-purpose flour with different Se treatments. Different lowercase letters of “a”–“l” indicate the significant (*p* < 0.05) differences between the bioaccessibility of Se in whole wheat with different Se treatments. Different uppercase letters of “A”–“F” indicate the significant (*p* < 0.05) differences between the bioaccessibility of Se in white all-purpose flour with different Se treatments.

More than 50% of Se was stored in edible parts such as grains, beans, and leafy vegetables as organic Se, when different species of Se(VI) or Se(IV) were applied (Hart et al., 2011; Lavu et al., 2012; Poblaciones et al., 2014; Muleya et al., 2021). This study found that SeMet (87–96%) and SeCys_2_ (4–13%) were the main Se species in wheat grains (93–100%; Figure 6), which is consistent with the findings of Poblaciones et al. (2014) and Hart et al. (2011). Similarly, Lu et al. (2018) showed that the main Se species of Se-enriched wheat was SeMet (44.2%), and SeCys_2_ (2.6%) and MeSeCys (0.3%). Muleya et al. (2021) also found that corn can effectively convert inorganic Se into organic Se, and more than 92% of Se exists as organic forms. Regardless of the species of exogenous Se, organic Se is often the main Se species measured in Se-enriched mushrooms and peanuts (Zhou et al., 2019; Luo et al., 2021).

Se(VI) is difficult to be converted into organic Se compared with Se(IV; Mazej et al., 2008). Theoretically, the ratio of organic Se to total Se in wheat grains treated with Se(VI) should be lower than that in plants treated with Se(IV; Wang et al., 2020). However, no significant difference was found in the percentage of organic Se in wheat (grains) after applying different species of Se in this study. While Eiche et al. (2015) found that the major Se species were SeMeCys (about 70%) and SeCys (about 30%) in the grains of wheat grown in natural Se-enriched areas through XANES (X-ray absorption near-side structure).

In general, most of the exogenous Se was accumulated in wheat leaves after foliar Se application (Wang et al., 2020). The percentage of Se(VI) in wheat grains also increased with the higher Se application rate in foliar Se treatments (Figure 6), and foliar Se(VI) application at pre-filling stage. The percentage of Se(VI) increased by 4% compared with pre-flowering stage in wheat grains (Figure 6). During the grouting stage, the migration efficiency of organic Se into the wheat grains was higher than that of inorganic Se, indicating that there was a higher inorganic Se content in the outer layer of the grain (Carey et al., 2012). Based upon these reported data, an in-depth understanding of the formation of various parts of the grain, such as bran, endosperm, and germ, is critical to fully understand the distribution of Se in whole grains.

### Effects of different flour yield on the bioaccessibility and content of Se in wheat

Recent studies have mainly focused on the bioaccessibility of Se in mushrooms (Zhou et al., 2019), grains and vegetables (Zhou et al., 2020), lettuce (Do et al., 2017), radish (Hu et al., 2020), potato (Dong et al., 2020), and maize (Zhou et al., 2020). Studies showed that Se-enriched *Pleurotus ostreatus* and *Pleurotus florida* had high Se bioaccessibility, which reached 70–92% (Zhou et al., 2019) and 60–80%, respectively (Bhatia et al., 2013), while the Se bioaccessibility in cereals was low (corn, 51%; rice, 65%; Jaiswal et al., 2012). This study found that the bioaccessibility of Se in whole wheat and white all-purpose flour of different Se treatments ranged from 6 to 38%. These results are consistent with the findings of Khanam and Platel (2016), who found that the bioaccessibility of Se in wheat grains ranged from 10 to 24%. In addition, Zhou et al. (2020) also showed that the Se bioaccessibility in maize was 8.8–22.5%. However, Lu et al. (2018) reported that the bioaccessibility of Se in Se-enriched wheat and soybeans reached 90%, corn and broccoli reached 80%, and cardamine hupingshanesis was 50%. These different percentages of Se bioaccessibility may have resulted from different Se application methods and types of crops.

Although the Se speciation in cereal crops has slightly different transformations (Muleya et al., 2021), no significant difference was found in Se bioaccessibility among them. The bioavailability of organic Se compounds is generally high (Muleya et al., 2021), due to the observation that organic Se is easily absorbed and utilized by humans (Gupta and Gupta, 2017). This study showed that organic Se was the main Se species in wheat grains (Figure 6). Consequently, the Se bioaccessibility in wheat should be higher. However, the observed low bioaccessibility of Se may be related to the bran component. The bioaccessibility of Se in white all-purpose flour was higher than that of whole wheat (Figure 5) confirmed that hypothesis. Reeves et al. (2007) found that the bioaccessibility of Se in refined wheat flour (mainly endosperm), wheat shorts (containing mainly germ), and wheat bran were 100, 85, and 60%, respectively. The low bioaccessibility of Se in bran is mainly because the Se-containing protein is wrapped by the non-digestible fiber in this component. Meanwhile, Khanam and Platel (2016) also found that the bioaccessibility of Se in intact legumes was lower than that of peeled legumes. Shen et al. (2019) showed that rice bran only accounts for about 7% of rice grain weight but contains about 14% of total Se in rice. Therefore, a considerable amount of Se may be lost in the process of removing wheat bran. Although the Se content of white all-purpose flour was lower than that of whole wheat, its bioaccessibility was high.

Different forms of applied exogenous Se also have different effects on the Se bioaccessibility of wheat. This study found that wheat treated with Se(VI) had higher Se bioaccessibility than Se(IV) treatments. Kápolna and Fodor (2007) also found that the bioaccessibility of Se in intestinal phase of Se-enriched green onions and leeks treated with Se (VI) was 80–90% and 12–28% with Se(IV) treatment. However, in the gastric phase of leek (*Allium ampeloprasum*), the Se bioaccessibility of Se(IV) treatment was slight higher than Se(VI) treatment (63 vs 56%), although this difference was not significant (*p* > 0.05; Lavu et al., 2012).

Regardless of the Se application methods, the Se bioaccessibility in intestinal phase of whole wheat and white all-purpose flour was higher than that in gastric phase. The results are consistent with the study of Lavu et al. (2012), which showed that the Se bioaccessibility in intestinal juice was 20% higher than gastric juice of Leek. The reasons may be as follows: (1) PBET is continuous (Toni et al., 2016), therefore, the Se bioaccessibility from the gastric phase to the intestinal phase is gradually accumulating; (2) in the intestinal phase, the existing digestive enzymes can hydrolyze polysaccharides, and then break down proteins into free amino acids and small molecular peptides, promoting the release of Se into the grains into the intestinal phase (Zhou et al., 2020). In this case, if a significant fraction of the bioaccessible Se has good chances to reach the colon, then it can be taken up by the microbial community and may also induce positive health effects. Further research is needed to evaluate whether this is actually the case.

## Conclusion

This research is the first systematic study conducted to explore Se bioassessibility in wheat Se fortified with different Se application methods. The wheat was separated into nine parts (sheath, first internode, first node, and rachis haven’t been systematically studied). The grain yield was the highest in plants treated with soil application at 2 mg kg^–1^ Se(IV), since Se(VI) has a higher Se bioavailability than Se(IV), there was an increased translocation of Se in wheat from the rachis to the grain. Both foliar and soil Se application can effectively increase the Se contents of wheat. The Se species applied to soil or to plant, application rates and growth stages applied, all influenced the Se content of wheat. Irrespective of Se application methods, the Se content of the first node was always higher than the first internode, indicating that the first node plays an important role in Se translocation in wheat. SeMet and SeCys_2_ were the main Se species in grains of wheat, indicating that wheat can efficiently convert applied inorganic Se into organic Se within the plant. In addition, flour milling process will cause losses of Se in wheat. The percentages of lost Se in white all-purpose flour were 12–68% higher compared with whole wheat. The Se bioaccessibility of whole wheat and white all-purpose flour with different Se treatments ranged from 6 to 38%, and white all-purpose flour had higher Se bioaccessibility than whole wheat. Future studies should also focus on the speciation changes, genotypes, and influence of the nodes on the mechanisms of Se translocation within wheat.

## Data availability statement

The original contributions presented in this study are included in the article/Supplementary material, further inquiries can be directed to the corresponding author.

## Author contributions

MW and DL created the hypothesis, objectives, outline the draft, and wrote the manuscript. FZ, NC, and PC performed the statistical analysis. YM, HZ, MQ, NL, YL, and LM performed the experiment. GB and DL revised the manuscript. All authors contributed to the article and approved the submitted version.
