# Supplementary material for: Soil and foliar selenium application: Impact on accumulation, speciation, and bioaccessibility of selenium in wheat (Triticum aestivum L.)
Source: Front Plant Sci. 2022 Sep 14;13:988627. doi: 10.3389/fpls.2022.988627 (PMC9516304; doi:10.3389/fpls.2022.988627)
Supplement: Supplementary file 1 [file Data_Sheet_1.zip › Graphical Abstract.docx]

**Graphical abstract for:**


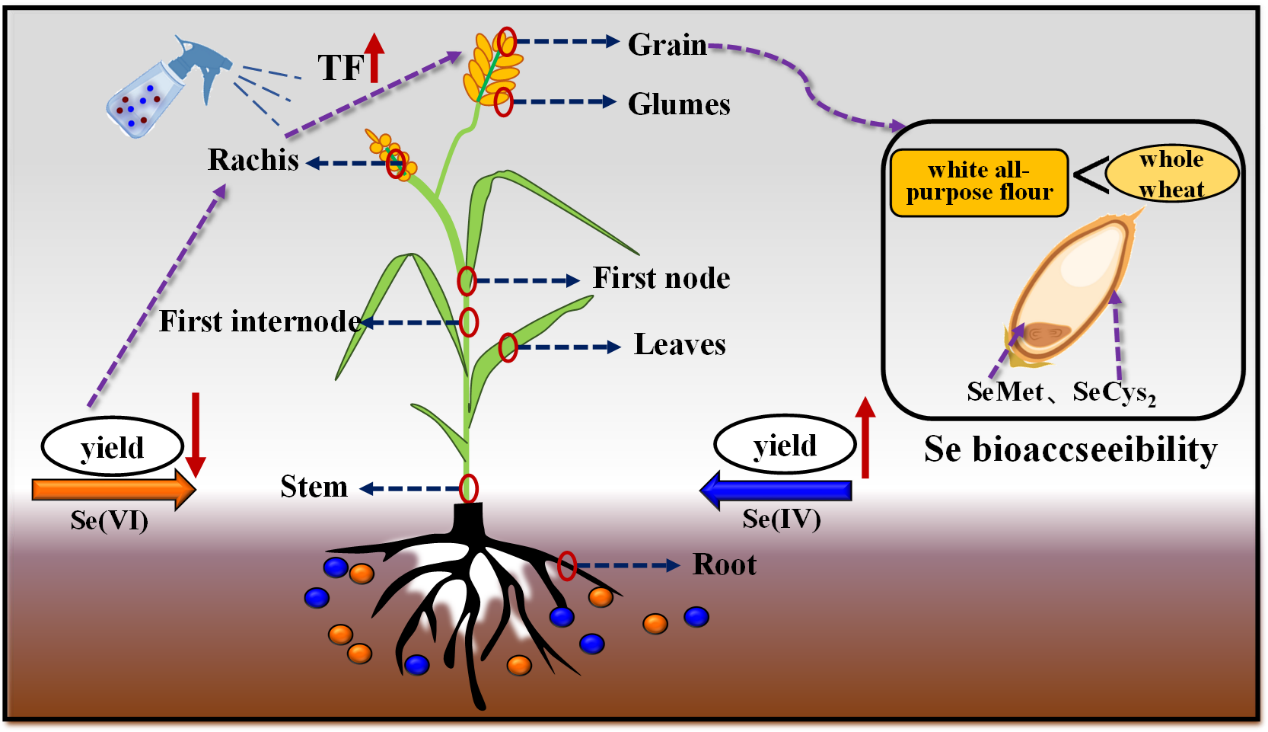
**Soil and foliar selenium application: Impact on accumulation, speciation, and bioaccessibility of selenium in wheat (*Triticum aestivum* L.)**
