# Supplementary material for: Soil and foliar selenium application: Impact on accumulation, speciation, and bioaccessibility of selenium in wheat (Triticum aestivum L.)
Source: Front Plant Sci. 2022 Sep 14;13:988627. doi: 10.3389/fpls.2022.988627 (PMC9516304; doi:10.3389/fpls.2022.988627)
Supplement: Supplementary file 1 [file Data_Sheet_1.zip › Supplementary Information.docx]

**Table S1** Biomass and yield of wheat under different foliar and soil Se applications.

**Figure S1** Effective ear number of wheat under different Se treatments of wheat under different Se treatments.

**Figure S2** The Se lost content and proportion in wheat flour under different Se treatments.

**Figure S3** Basic growth indicators of wheat under different Se treatments.

**Table S1** Biomass and yield of wheat under different foliar and soil Se applications.

| Application methods | Se species | Growth stages | Se application rates | Grain yield (g pot^-1^) | Biomass (g pot^-1^) |
| --- | --- | --- | --- | --- | --- |
|  |  |  |  |  |  |
| CK | | | | 20.33 ± 0.22 Aab | 41.10 ± 0.34 Ab |
| Soil application | Se(IV) |  | 0.5 mg kg^-1^ | 19.41 ± 0.21 b | 44.37 ± 0.43 ab |
|  |  |  | 1 mg kg^-1^ | 19.42 ± 1.14 b | 44.87 ± 4.05 ab |
|  |  |  | 2 mg kg^-1^ | 21.62 ± 1.38 a | 46.03 ± 1.83 a |
|  | Se(VI) |  | 0.5 mg kg^-1^ | 19.48 ± 0.42 A | 40.11 ± 1.40 A |
|  |  |  | 1 mg kg^-1^ | 15.24 ± 0.73 B | 34.06 ± 2.33 B |
|  |  |  | 2 mg kg^-1^ | 7.65 ± 0.45 C | 16.69 ± 2.15 C |
| Foliar application | Se(IV) | F1 | 5 mg L^-1^ | 19.77 ± 0.17 ab | 38.70 ± 0.90 a |
|  |  |  | 10 mg L^-1^ | 19.47 ± 0.29 ab | 39.06 ± 2.82 a |
|  |  |  | 20 mg L^-1^ | 18.19 ± 0.12 c | 38.74 ± 0.49 a |
|  |  | F2 | 5 mg L^-1^ | 19.34 ± 0.46 b | 38.27 ± 3.55 a |
|  |  |  | 10 mg L^-1^ | 19.22 ± 0.70 b | 38.65 ± 0.49 a |
|  |  |  | 20 mg L^-1^ | 18.22 ± 0.91 c | 39.59 ± 3.02 a |
|  | Se(VI) | F1 | 5 mg L^-1^ | 20.28 ± 0.77 A | 41.52 ± 2.76 A |
|  |  |  | 10 mg L^-1^ | 19.67 ± 0.11 AB | 39.76 ± 1.89 A |
|  |  |  | 20 mg L^-1^ | 18.85 ± 0.23 CD | 38.92 ± 2.10 A |
|  |  | F2 | 5 mg L^-1^ | 19.29 ± 0.20 BC | 38.62 ± 0.36 A |
|  |  |  | 10 mg L^-1^ | 19.65 ± 0.21 AB | 39.25 ± 1.69 A |
|  |  |  | 20 mg L^-1^ | 18.50 ±0.35 D | 39.64 ± 1.06 A |
| ANOVA (*F* value) | | | |  |  |
| Se sources (SS) | | | | ** | ** |
| Se rates (SR) | | | | ** | ** |
| Se rates (R) | | | | ** | NS |
| Se sources (S) | | | | NS | NS |
| Growth stages (G) | | | | NS | NS |
| S × R | | | | NS | NS |
| S × G | | | | NS | NS |
| G × R | | | | NS | NS |
| SS × SR | | | | ** | ** |
| S × G × R | | | | NS | NS |

Note: F1 represents pre-flowering stage, F2 represents pre-filling stage. CK=control. Different lowercase letters “a-d” indicate the difference of Se(IV) treatments (*p* < 0.05). Different capital letters “A-D” denote the difference between Se(VI) treatments (*p* < 0.05). “**” indicates a significant correlation of 99%, “*” indicates a significant of 95% correlation, and “NS” indicates no correlation. “SS” represents Se sources in soil Se application. “SS” represents Se sources in soil Se application. “SR” represents Se rates in soil Se application. “R” represents Se rates in foliar Se application. “S” represents Se sources in foliar Se application. “G” represents growth stages of wheat in foliar Se application.

**
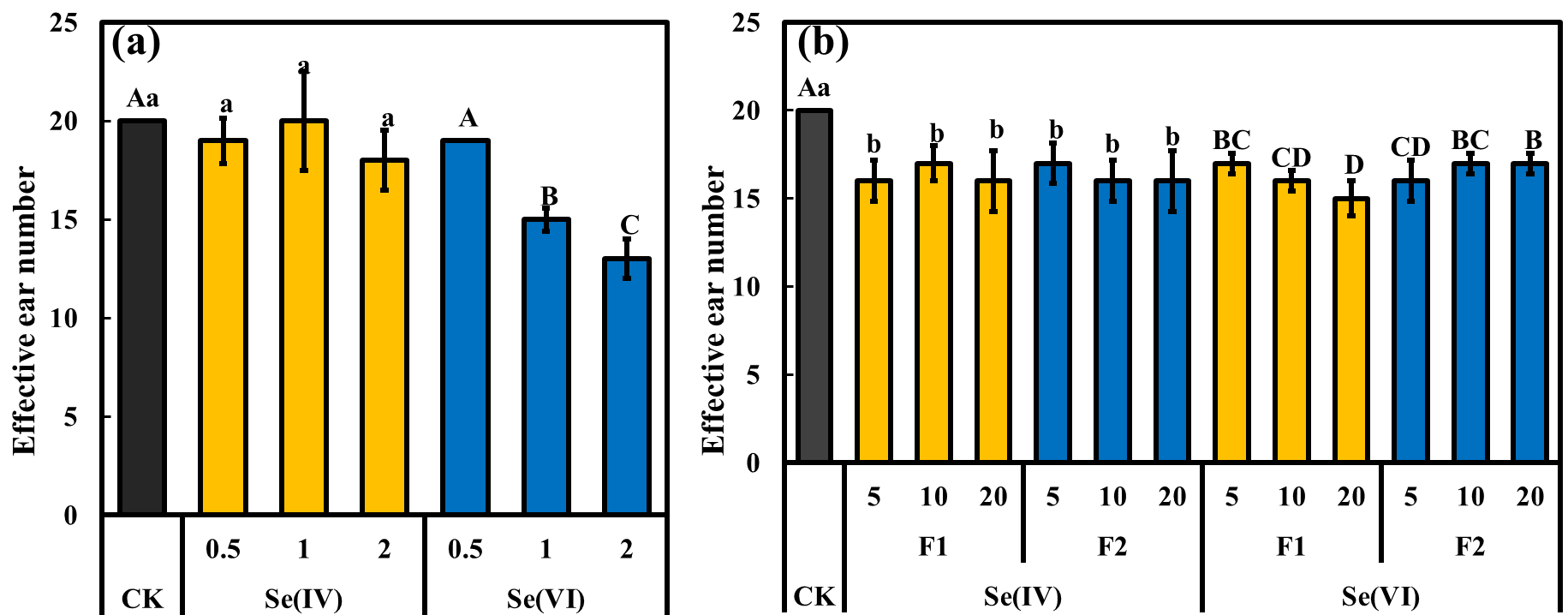
****Figure S1** Effective ear number of wheat under different Se treatments.

Note: (a) and (b) represent the effective ear number of the wheat of soil and foliar Se application, respectively. F1 represents pre-flowering stage, F2 represents pre-filling stage. Different lowercase letters “a-c” indicate the difference of Se(IV) treatments (*p* < 0.05). Different capital letters “A~D” denote the difference between Se(VI) treatments (*p* < 0.05).

**Figure S2** The Se lost content and proportion in wheat flour under different Se treatments.


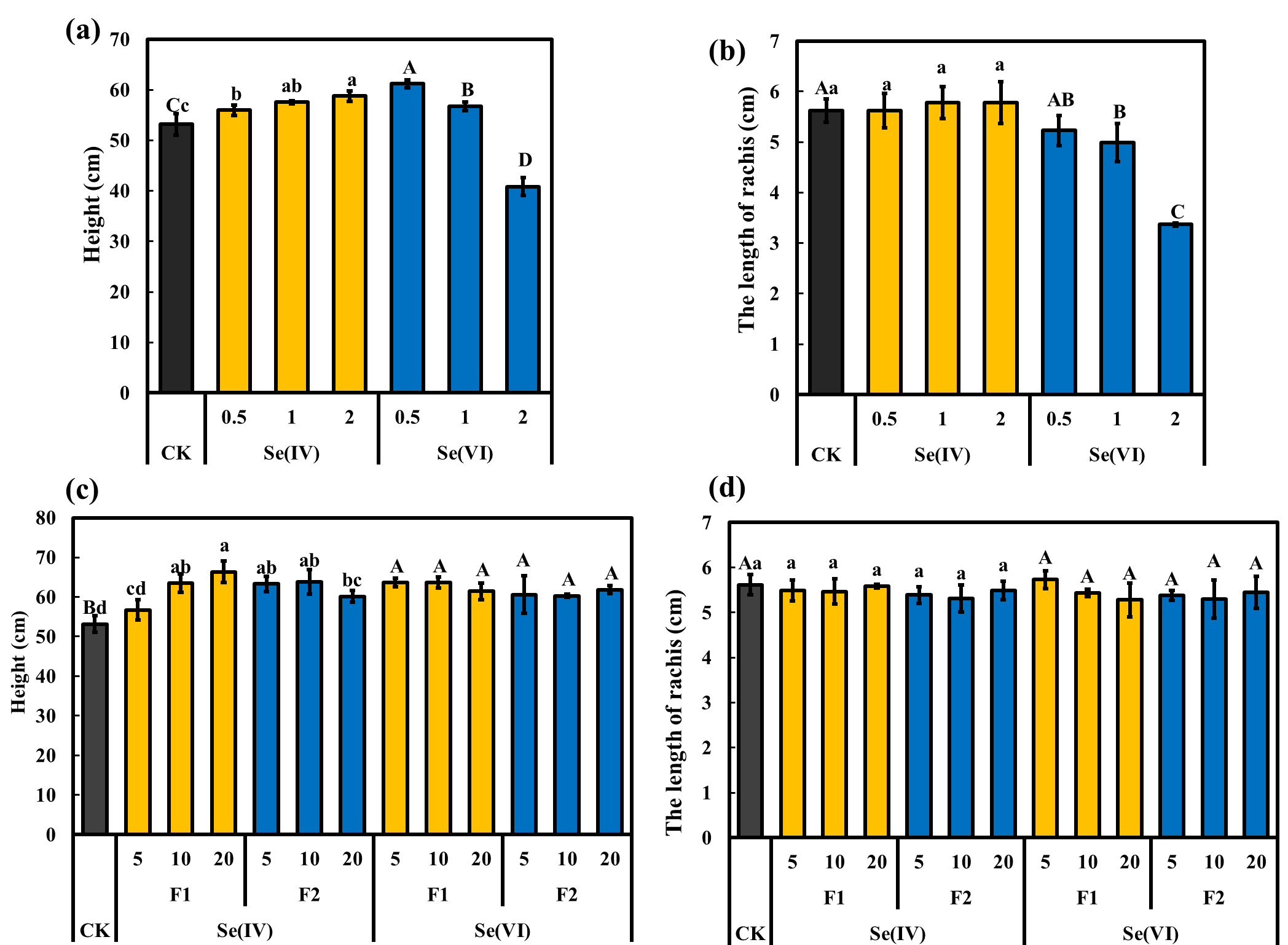


**Figure S3** Basic growth indicators of wheat under different Se treatments.

Note: (a) and (c) represent the height of the wheat of soil and foliar Se application, respectively; (b) and (d) represent the length of the rachis of the wheat of soil and foliar Se application, respectively. F1 represents pre-flowering stage, F2 represents pre-filling stage. Different lowercase letters “a-c” indicate the difference of Se(IV) treatments (*p* < 0.05). Different capital letters “A-D” denote the difference between Se(VI) treatments (*p* < 0.05).
